# Exclusive expression of MeCP2 in the nervous system distinguishes between brain and peripheral Rett syndrome-like phenotypes

**DOI:** 10.1093/hmg/ddw269

**Published:** 2016-08-09

**Authors:** Paul D. Ross, Jacky Guy, Jim Selfridge, Bushra Kamal, Noha Bahey, K. Elizabeth Tanner, Thomas H. Gillingwater, Ross A. Jones, Christopher M. Loughrey, Charlotte S. McCarroll, Mark E.S. Bailey, Adrian Bird, Stuart Cobb

**Affiliations:** 1 Institute of Neuroscience and Psychology, College of Medical, Veterinary & Life Sciences, University of Glasgow, Glasgow, UK; 2 Wellcome Trust Centre for Cell Biology, University of Edinburgh, Michael Swann Building, Edinburgh, UK; 3 Histology Department, Faculty of Medicine, Tanta University, Tanta, Egypt; 4 School of Engineering, University of Glasgow, Glasgow, UK; 5 Edinburgh Medical School: Biomedical Sciences, University of Edinburgh, Hugh Robson Building, Edinburgh, UK; 6 Institute of Cardiovascular and Medical Sciences, University of Glasgow, Glasgow, UK and; 7 School of Life Sciences, College of Medical, Veterinary & Life Sciences, University of Glasgow, Glasgow, UK

## Abstract

Rett syndrome (RTT) is a severe genetic disorder resulting from mutations in the X-linked *MECP2* gene. MeCP2 protein is highly expressed in the nervous system and deficiency in the mouse central nervous system alone recapitulates many features of the disorder. This suggests that RTT is primarily a neurological disorder, although the protein is reportedly widely expressed throughout the body. To determine whether aspects of the RTT phenotype that originate in non-neuronal tissues might have been overlooked, we generated mice in which *Mecp2* remains at near normal levels in the nervous system, but is severely depleted elsewhere. Comparison of these mice with wild type and globally MeCP2-deficient mice showed that the majority of RTT-associated behavioural, sensorimotor, gait and autonomic (respiratory and cardiac) phenotypes are absent. Specific peripheral phenotypes were observed, however, most notably hypo-activity, exercise fatigue and bone abnormalities. Our results confirm that the brain should be the primary target for potential RTT therapies, but also strongly suggest that some less extreme but clinically significant aspects of the disorder arise independently of defects in the nervous system.

## Introduction

Rett Syndrome (RTT) is an X-linked genetic disorder that is a leading cause of intellectual disability in girls and women (1). Diagnostic features of typical RTT include a highly characteristic developmental regression involving loss or impairment of mobility and loss of learnt speech and skilled intentional hand movements, accompanied by stereotypic hand movement automatisms. Associated features, such as microcephaly, respiratory/autonomic abnormalities, seizures, growth deficits and early hypotonia are highly prevalent (1,2). Later in childhood, RTT patients often develop prominent skeletal signs including severe scoliosis, early osteoporosis and a propensity to suffer low-impact fractures and hip deformities (3–5). In the vast majority of cases RTT is caused by *de novo* mutations in the *MECP2* gene, which encodes methyl-CpG binding protein 2 (MeCP2) (6), an abundant nuclear protein that is considered to be important in chromatin-level regulation of transcription (7). MeCP2 is thought to mediate transcriptional inhibition by binding to methylated CpG and CpA dinucleotides in the genome (8–10) and recruiting co-repressor complexes (11–14). Other reports suggest MeCP2 may also function as an activator of transcription (15) amongst other functions (7).

Mouse models of RTT have been developed that typically recapitulate many of the characteristic features of the human disorder and allow the underlying pathogenic mechanisms involved to be investigated (16,17). Two studies reported that deletion of *Mecp2* specifically in the nervous system results in the full range of RTT-like phenotypes (16,17), suggesting that the disorder is primarily neuronal in origin. These studies only investigated gross aspects of the phenotype, however, such as body weight, survival and brain size. It is possible that the shortened lifespan of null model masks phenotypes that are either subtle or delayed in their onset. In addition, more recent studies have identified a number of novel phenotypes, including cardiovascular abnormalities (18,19), lung abnormalities (20), bone and skeletal muscle defects (21–23) and altered cholesterol biosynthesis (24–26). MeCP2-deficiency outside the nervous system (that is, in the “periphery”) potentially contributes to these phenotypes. MeCP2 is widely expressed, with high levels reported in specific cell types of heart, lung and various other peripheral tissues (27–28). These findings highlight our ignorance of the relative contributions of MeCP2-insufficiency in the nervous system versus peripheral tissues to the pathogenesis of MeCP2 disorders.

To address this knowledge gap, we generated a mouse model in which *Mecp2* is silenced in peripheral tissues, but reactivated prenatally at near normal levels within the nervous system. Using this model, we investigated the peripheral contribution to the major RTT-like phenotypes, as well as less prominent aspects of the disorder. We also carried out in-depth analysis of aspects of tissue function using metabolic and other tests to seek previously undetected phenotypes. The experiments reveal that the major features that characterise RTT result from lack of MeCP2 in the nervous system. Lack of MeCP2 in the periphery, however, resulted in phenotypes that match specific clinical features associated with RTT, including reduced stamina and bone abnormalities. Thus, while the nervous system should be the major focus for targeting future RTT therapies, our results suggest that a subset of phenotypes may be peripheral in origin and may therefore benefit from systemic level interventions.

## Results

### Levels of native MeCP2 in the nervous system and peripheral tissues

**Figure 1.**
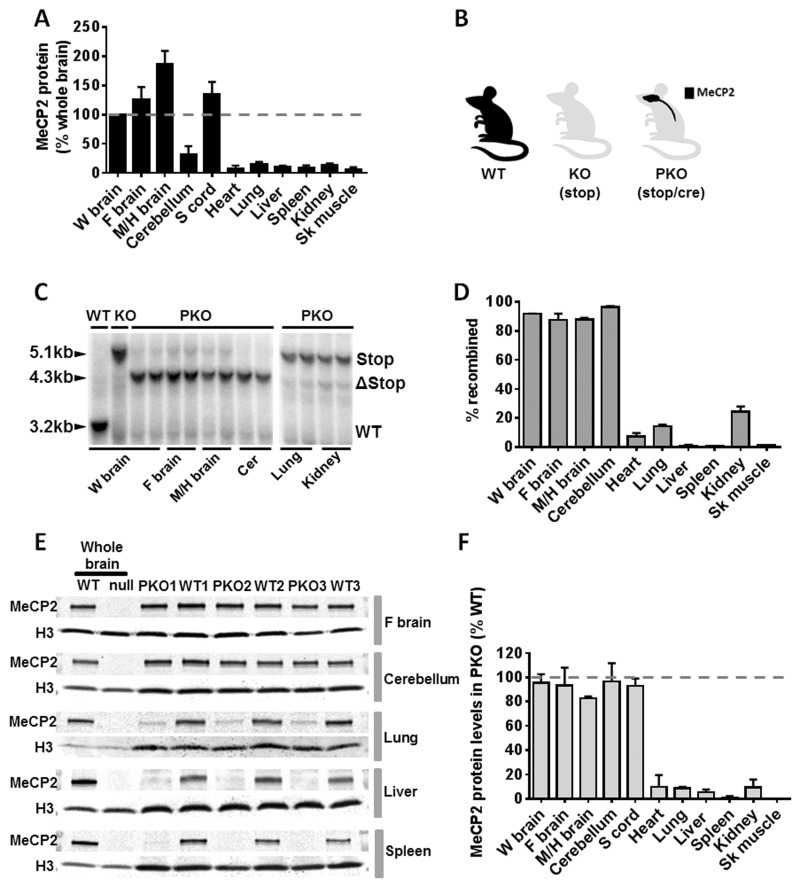
Generation of a CNS rescue or ‘peripheral knockout’ mouse. **(A)** Plot showing levels of native MeCP2 protein relative to whole brain levels (and standardised to histone H3 levels) in wild type (WT) mice as revealed by quantitative immunoblot (mean ± S.D., *n =* 3). **(B)** Cartoon showing MeCP2 expression profile in three experimental cohorts of mice including WT, knockout (KO) in which *Mecp2* is silenced globally using a stop cassette, and a peripheral knockout (PKO) mouse in which *Mecp2* is silenced in the peripheral tissues but reactivated in the nervous system using nestin-cre mediated recombination of an Mecp2-stop allele. **(C)** Representative Southern blots showing recombination of the Stop/y allele (5.1 kb) to delete the Stop cassette (open triangle, 4.3 kb) in PKO mice. **(D)** Plot showing recombination efficiency in brain and peripheral tissues as revealed by Southern blot analysis (*n =* 2–4 mice; mean ± S.D.). **(E)** Representative quantitative western blots showing MeCP2 expression in WT, PKO and null mice. WT whole brain reference samples were used to enable comparison of MeCP2 levels between different gels and different tissues. Histone H3 was used as a loading control. **(F)** MeCP2 protein levels in PKO mice relative to WT levels across representative tissues (mean ± S.D., *n =* 3). Abbreviations: W brain, whole brain; F brain, forebrain; M/H brain, Mid/hindbrain; Sk muscle, skeletal muscle; Cer, Cerebellum.

We first measured levels of MeCP2 expression in several mouse tissues in comparison with brain, using quantitative western blotting of whole tissue homogenates. As an internal calibration control, we chose histone H3, as this protein is a component of the nucleosome core that is present in a constant ratio with DNA and therefore cell number in somatic tissues. The results of biological triplicate experiments gave consistent results showing that levels in a variety of peripheral tissues are approximately an order of magnitude lower per average cell than in the nervous system (Fig. 1A). Levels in the forebrain, mid- and hind-brain and spinal cord are above the average level for whole brain, but cerebellum is significantly lower than other tested brain regions. It is likely that average MeCP2 abundance in the brain is reduced by the contribution of cerebellar granule cells, which are the most abundant and among the smallest neurons in the brain. Our findings are at variance with a previous estimate of MeCP2 abundance in mouse tissues, which concluded that MeCP2 is most highly expressed in lung and somewhat lower in spleen and brain (29). In that study protein levels were normalised to glyceraldehyde 3-phosphate dehydrogenase (GAPDH) and alpha-tubulin, both of which are cytoplasmic proteins whose abundance in cells of different sizes is unlikely to be constant. Histone H3 has greater validity as a comparator, as nucleosomes uniformly coat the genome with a periodicity close to 200 base pairs in multiple cell types and organisms. Consistent with this generalisation, the histone:DNA ratio has long been known to be close to 1:1 for chromatin from diverse sources (30,31). These observations, together with previous evidence that MeCP2 is extremely highly expressed in neurons (32), argue that the present findings accurately reflect low levels of MeCP2 protein in tissues outside the nervous system.

### Generation of *Mecp2* peripheral knockout mice

To generate mice in which MeCP2 is selectively present in the nervous system, but largely absent in the periphery, we utilised a mouse line in which *Mecp2* has been silenced by a stop cassette targeted to the *Mecp2* locus (33). It was shown previously that the stop cassette causes a >95% reduction in MeCP2 levels and is functionally almost equivalent to a full knockout allele (34). The stop cassette in this line, referred to as KO, is flanked by loxP sites whose deletion by cre results in efficient restoration of normal expression and reverses phenotypic defects (33,34). To generate a ‘peripheral knockout’ (PKO; Fig. 1B), we reactivated the *Mecp2* gene specifically in neurons and glia of the nervous system (Fig. 1B) by crossing with a mouse line expressing cre from the nestin promoter (35). Southern blot analysis revealed a high recombination frequency in the brain of PKO mice (≥90%), but very low levels of recombination (below 15%) in all other tissues tested (Fig. 1C and D) with the exception of kidney (24%). These values agree with the previously reported expression patterns of nestin-cre (35,36). This was confirmed at the protein level by quantitative western blots (Fig. 1E and F) and anti-MeCP2 immunolabelling of tissue sections (Supplementary Material, Fig. S1), which showed near wildtype levels of MeCP2 in the brain and spinal cord, but greatly reduced levels in other tissues. We conclude that the PKO mouse retains MeCP2 expression in the vast majority of cells in the nervous system, whereas only a small minority of cells in peripheral tissues express the protein.

### Nestin-cre-mediated rescue of brain MeCP2 prevents the onset of overt RTT-like phenotypes

**Figure 2.**
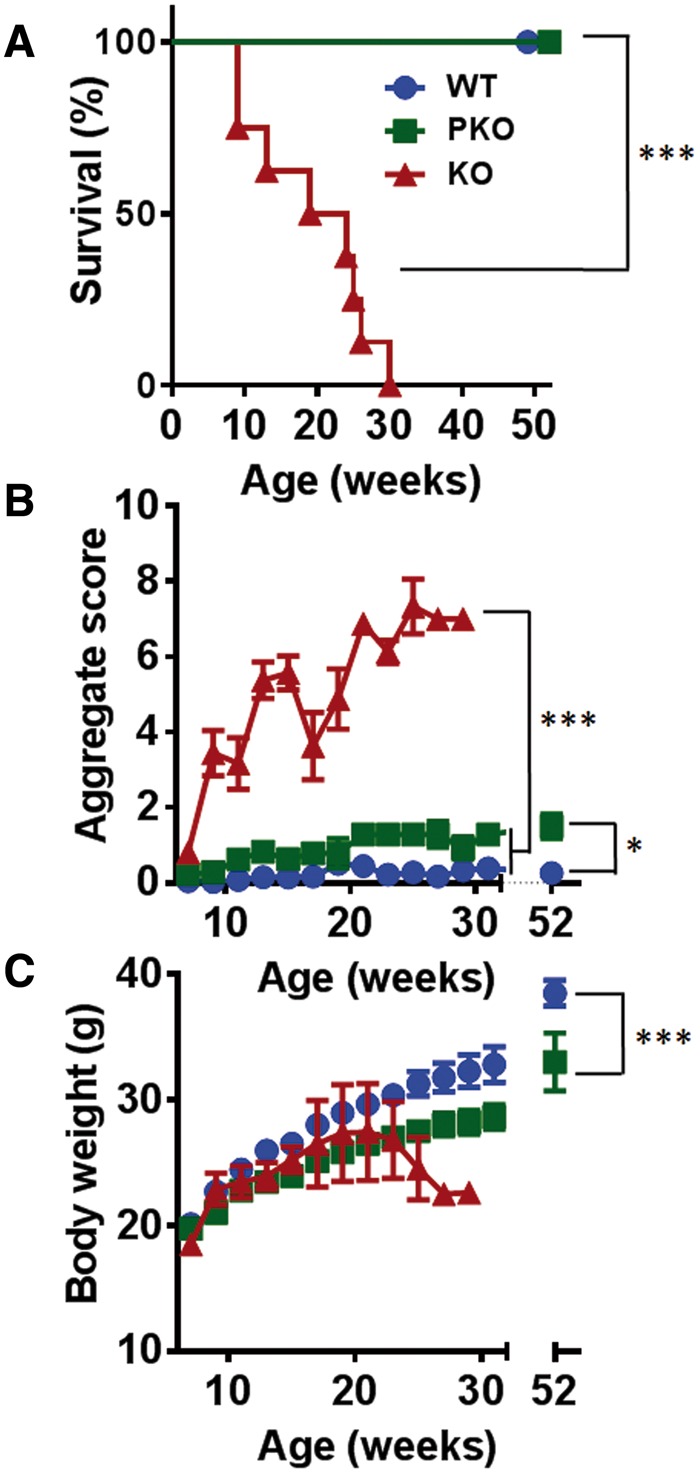
Normal survival and absence of RTT-like signs in peripheral KO mice. (A) Survival plot showing normal survival of WT (blue circles, *n =* 17) and PKO mice (green squares, *n =* 7) compared to reduced lifespan in KO mice (red triangles, *n =* 8). The median survival period was significantly reduced in KO mice (***P < 0.001, log-rank test). **(B)** Plot showing aggregate phenotype severity (mean ± SEM) based on an established observational scoring system. There was a highly significant difference in score between WT and PKO animals from 8 weeks onwards when compared to KO (***P <* *0.001, Kruskal-Wallis test with Dunn’s post hoc analysis). No significant difference was seen between WT and PKO at the time of behavioural testing (15 weeks) but these groups differed significantly at 1 year (*P < 0.05). **(C)** Plot showing body weight changes over time (mean ± SEM). No significant differences were seen between genotypes at the time of behavioural testing (15 weeks; *P* > 0.05, one-way ANOVA) but PKO mice had significantly lower bodyweight than WT mice when compared at 1 year (***P < 0.001, student’s unpaired t-test). In B) and C) group sizes at the start of the experiment are as given in A).

KO mice survived to a median of 150 days, consistent with previous reports (33). In contrast, PKO and WT cohorts were fully viable and fertile over the 52-week test period (Fig. 2A). Indeed, a subgroup of PKO mice were maintained for 2 years with no deaths recorded. Mice were assessed weekly for RTT-like signs using an established observational scoring system (33). Phenotype severity increased over time in the KO mice (reflecting impaired locomotion and breathing, tremor and poor general condition, see Methods) and differed significantly from that of both WT and PKO animals (*P* <* *0.01) (Fig. 2B). WT and PKO mice did not show a significant difference over the initial 30 week scoring period, but when assessed again at one-year-old PKO mice showed a modest reduction in activity and mild gait abnormalities compared to WT (*P* < 0.05; Fig. 2B). Bodyweight was indistinguishable between PKO and WT genotypes at 14–16 weeks, but at 1 year, PKO mice weighed somewhat less than WT mice (Fig. 2C; mean bodyweight: WT = 38.5 ± 1.0 g; PKO = 33.0 ± 2.3 g; *P* < 0.05). These minor differences were not affected by the presence or absence of the cre transgene in WT control mice, as a comparison of weights and phenotypic scores between cre-positive and cre-negative mice showed no significant differences (Supplementary Material, Fig. S2).

In addition to gross severity and bodyweight, mice from each genotype were examined for routine blood biochemistry (Supplementary Material, Table S1) and histopathological changes across a panel of tissues and organs (Supplementary Material, Table S2). Blood serum measures showed modest changes in a small number of markers in the KO group, but there were no significant differences between PKO and WT samples across all measures. Histopathological evaluation revealed no gross structural or histopathological changes for the majority of tissues examined. Kidney sections from KO and PKO mice revealed mild to moderate vacuolation, however, potentially indicative of lipid accumulation.

### PKO mice show mild hypoactivity but absence of behavioural or gait defects

**Figure 3.**
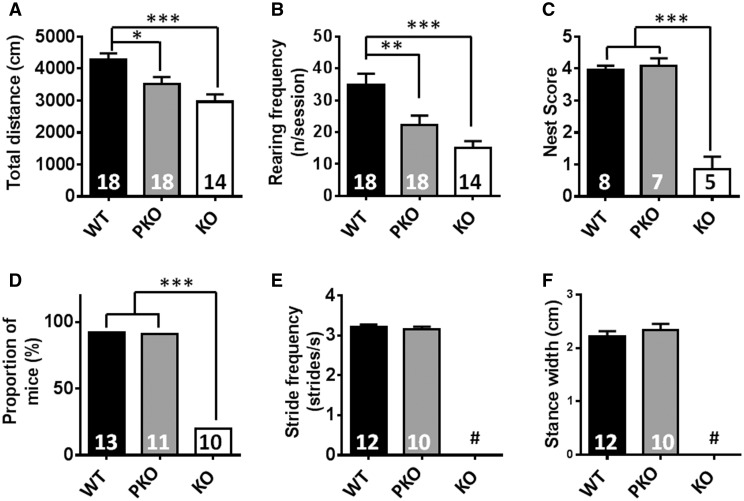
PKO mice show mild hypoactivity but absence of behavioural or gait defects. (**A–B**) Spontaneous motor and exploratory activity assessed using open-field. Results show (A) total distance moved and (B) number of rearing events/session. There was a significant difference from WT in both PKO and KO mice. (**C**) Nesting behaviour was normal in PKO mice but impaired in KO mice. (**D–F**) Gait assessed using motorised treadmill. Results show (D) Proportion of mice capable of performing to criterion (E) stride frequency and (F) stance width. All data other than (D) are mean ± SEM. Numbers of animals per genotype are shown within each bar. Groups were compared using one-way ANOVA with Tukey’s *post hoc* analysis. # indicates not determined due to mice being unable to perform to criterion. *P < 0.05, **P < 0.01, ***P < 0.001.

Having established that PKO mice did not differ markedly from WT counterparts at a gross level, we next conducted a fine-grained phenotypic analysis to detect archetypal RTT-like features, including behavioural, locomotor and respiratory phenotypes. This analysis was conducted at 14–16 weeks of age, at which time the surviving KO mice had become overtly symptomatic but could still complete most physiological and behavioural testing paradigms. A previous study has shown that the nestin-cre transgenic line used in this study displays a mild metabolic and behavioural phenotype (37). To control for this potential confounding factor, all WT control mice used for the behavioural analysis also contained the nestin-cre transgene. In order to assess spontaneous movement, whose loss is prominent in all *Mecp2* knockout and knock-in models (11,17,38,39), mice were monitored whilst ambulating freely in an open-field arena. KO and PKO mice showed a reduction in both the distance moved during the trial and in the amount of time spent rearing (regarded as a measure of exploratory behaviour) compared to WT animals, although the difference was more modest in PKO animals (Fig. 3A and B, distance moved in 10 min: WT = 4242 ± 167; PKO = 3523 ± 215; KO = 2963 ± 230 cm; rearing instances per session; WT = 35.0 ± 3.5; PKO = 22.4 ± 2.9; KO = 15.0 ± 2.2; *P* < 0.05). We also assessed nest-building (40), as failure to utilise nesting materials is associated with cognitive and motor impairments (41–43). In agreement with previous studies of *Mecp2*-null mice (44), KO mice showed a profound reduction in nest-building score compared to WT (Fig. 3C**;** nesting score: KO = 0.90 ± 0.15; PKO = 4.10 ± 0.25; WT = 4.00 ± 0.40; *P* <* *0.001). In contrast, PKO mice did not differ from WT (*P* > 0.05).

Hand stereotypies and gait abnormalities constitute major diagnostic criteria in RTT (1). Gait was therefore assessed using a treadmill-based system. Previous studies using this approach (45) have shown that KO mice develop a range of characteristic gait defects that increase in severity between 4 and 10 weeks of age. We found that older symptomatic KO animals (14–16 weeks) were largely incapable of continuous running (10 cm/s) on a treadmill (Fig. 3D). PKO mice, on the other hand, displayed no differences in stride frequency (Fig. 3E), stance width (Fig. 3F) or a range of other gait parameters (Supplementary Material, Table S2) compared to WT animals. These results indicate that absence of MeCP2 from peripheral tissue leads to modest reductions in overall locomotor activity without significantly affecting gait.

### PKO mice show no difference in balance but marked deficiency in exercise capacity

**Figure 4.**
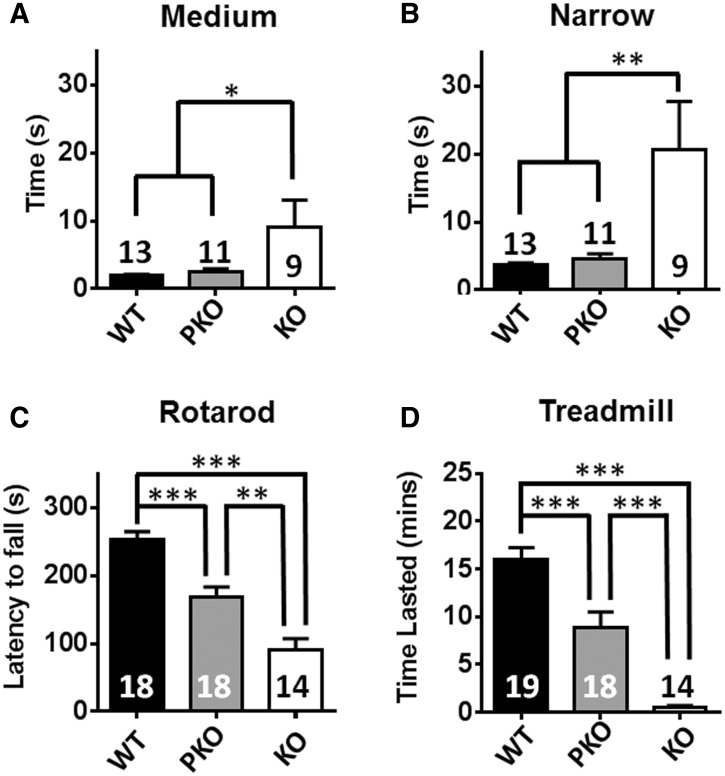
PKO mice show no difference in balance but marked deficiency in exercise capacity. (**A–B**) Balance beam task showing time to traverse medium (A) and narrow (B) beams. (**C**) Rotarod performance. (**D**) Exercise capacity measured using an elevated treadmill, with a steadily increasing speed. Results show time lasted on treadmill. Plots show mean ± S.E.M. Number of animals per genotype are shown within each bar. Groups were compared using one-way ANOVA and Tukey’s *post hoc analysis*. *P < 0.05, **P < 0.01, ***P < 0.001.

To further examine the motor function, balance and coordination were assessed using an inclined beam and rotarod. In the beam test, KO mice showed deficits on both medium (time to traverse 11 mm-wide beam: WT = 1.9 ± 0.3; PKO = 2.5 ± 0.4; KO = 9.1 ± 4.0 s; *P* < 0.05) and narrow beams (time to traverse 5 mm-wide beam: WT = 3.7 ± 0.3 s; PKO = 4.6 ± 0.8 s; KO =  20.6 ± 7.2 s; *P* < 0. 01) whereas PKO mice were not different from WT (Fig. 4A and B). The rotarod test also revealed a markedly reduced performance in KO mice compared to both WT and PKO littermates (Fig. 4C; latency to fall: WT = 243.5 ± 11.5; PKO = 168 ± 14.9; KO = 91.5 ± 16.1 s; *P* < 0.001). In contrast to the balance beam data, however, rotarod detected a reduction in performance in the PKO mice compared to WT (*P* < 0.001). The accelerating rotarod test is commonly used to assess balance and coordination defects, but is also sensitive to endurance fatigue. Since PKO mice did not display overt coordination and balance defects in the beam test, we hypothesised that their reduced rotarod performance was due to exercise fatigue associated with the challenging nature of the accelerating rotarod task. In order to test this hypothesis, mice were subjected to an inclined treadmill task commonly used to test exercise fatigue (46). A mild aversive stimulus ensures that mice carry out the task to their true capacity and helps eliminate motivational state as a confounding factor. KO mice showed a profoundly impaired performance compared to both PKO and WT mice (Fig. 4D; time sustained on treadmill; WT = 16.5 ± 1.3; PKO =  8.7 ± 1.6; KO = 0.5 ± 0.2 min; *P* < 0.001), but once again PKO mice performed significantly less well than WT (*P* < 0.001). These results, in combination with the rotarod data, suggest that absence of MeCP2 from peripheral tissues leads to a marked reduction in exercise capacity/increased vulnerability to fatigue, which is not due to reduced motivation.

### RTT-like respiratory phenotypes are absent in PKO mice

**Figure 5.**
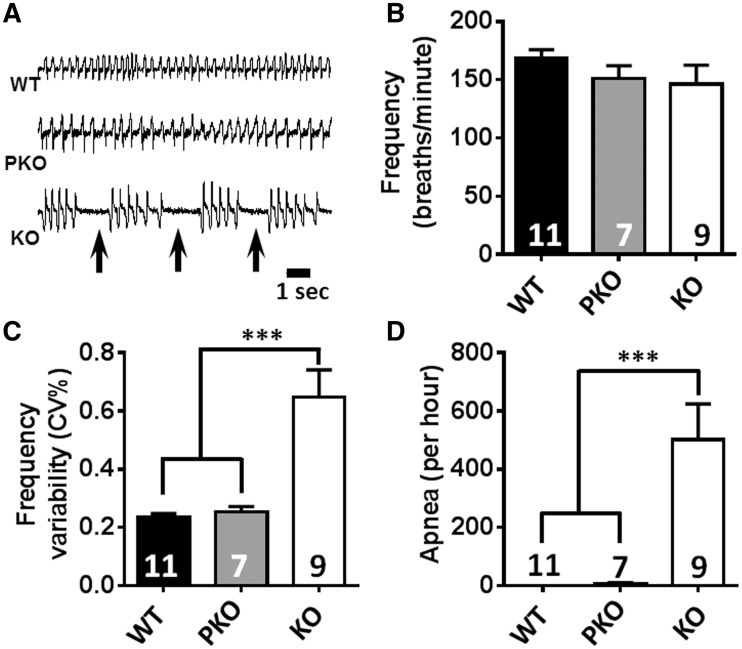
RTT-like respiratory phenotypes are absent in PKO mice. **(A)** Representative whole-body plethysmograph traces showing regular and erratic breathing patterns/apnoeas (arrows) in WT, stop-cre mice and in stop mice, respectively. **(B)** breathing frequency at rest, **(C)** breathing frequency variability and **(D)** apnoea frequency. In (B–D), data are plotted as mean values ± S.E.M. Numbers of animals per genotype are shown within each bar. Groups were compared using one-way ANOVA and Tukey’s *post hoc* comparisons. ***P < 0.001.

Abnormal breathing patterns, including breath holding and apnoea, are common features of RTT (47) and are also observed in *Mecp2* KO mice (34,48–51). They are typically attributed to disruption of autonomic or brainstem function, but as MeCP2 protein is expressed in lung, albeit at modest levels, and clinical studies suggest that pulmonary lesions are common in RTT (20), we looked for a peripheral contribution to respiratory pathologies. We initially assessed conscious resting breathing function using whole-body plethysmography. Respiratory traces (Fig. 5A) were analysed for two prominent characteristics of the RTT breathing phenotype: breath frequency/irregularity and the presence of apnoeas. No differences were observed for breathing frequency between the three groups (Fig. 5B), but analysis of the coefficient of variability (CV, %) of respiratory frequency (Fig. 5C) revealed that KO animals showed a highly irregular breathing pattern (mean CV = 0.65 ± 0.09%) compared to both WT and PKO animals (*P* < 0.001). In contrast, PKO animals displayed a highly regular breathing pattern (CV = 0.26 ± 0.02%) that was indistinguishable from WT (CV = 0.24 ± 0.01%). Similarly, WT and PKO mice showed negligible occurrence of apnoeas (Fig. 5D; Apnoea number: WT = 0; PKO = 7 ± 4.61/h; *P* > 0.05) whilst KO mice showed a very high incidence of such events (mean = 501 ± 123.7/h) that differed from both WT and PKO mice (*P* < 0.001). These results suggest that the absence of MeCP2 from peripheral tissues does not lead to the respiratory dysfunction typically present in *Mecp2*-null mice. Additionally, histopathological examination of lung tissue biopsies (three per genotype) revealed no gross structural abnormalities, inflammation or other pathological signs in any of the genotypes (Supplementary Material, Table S2).

### Echocardiography results show absence of cardiovascular phenotypes in PKO mice

**Figure 6.**
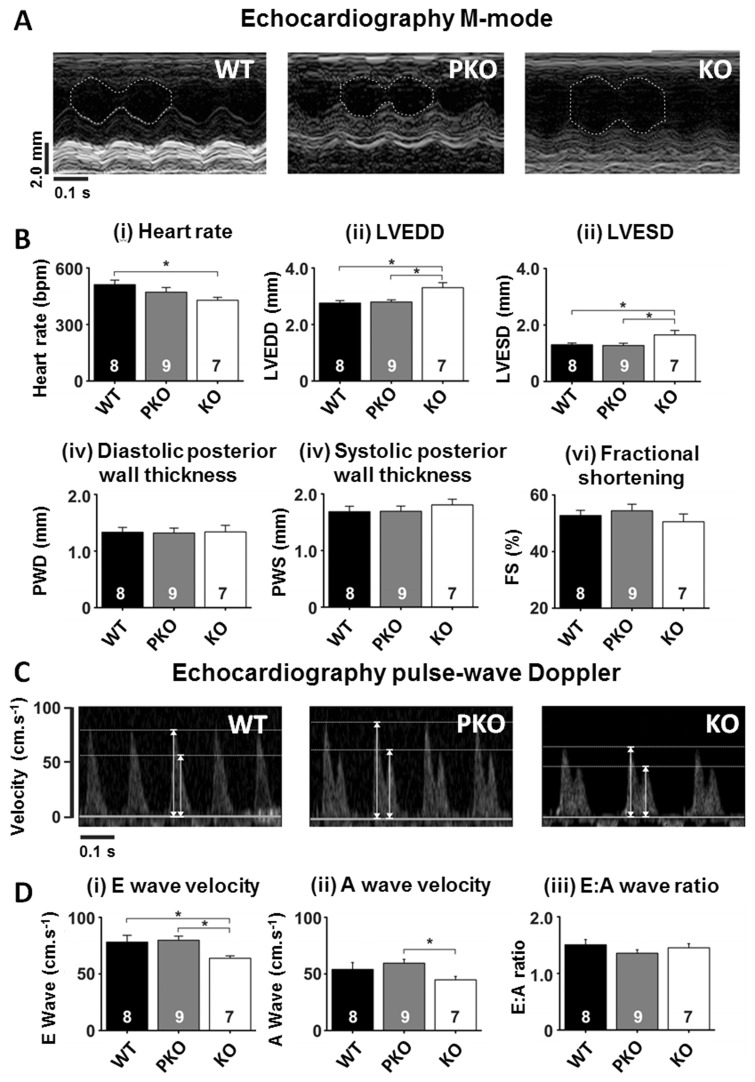
Echocardiography parameters unchanged in PKO mice. **(A)** Example M-mode images from (i) WT, (ii) PKO and (iii) KO mice. The left ventricular internal diameter is indicated in each image by the white dashed line. **(B)** Mean ± SEM for M-mode measured parameters. **(C)** Example pulse-wave Doppler images from each of the experimental and control groups. Arrows indicate peak heights for both E (early diastolic filling) and A (atrial contribution to diastolic filling) waves. **(D)** Mean ± SEM for pulse-wave Doppler measured parameters. Numbers of animals per genotype are shown within each bar. Groups were compared using one-way ANOVA and Tukey’s *post hoc* comparisons. *P < 0.05.

Having assessed core phenotypes that define RTT, we next examined other peripheral tissue functions that are known to be altered in RTT. Echocardiography revealed defects in heart rate and cardiac contractile function in KO mice that were absent in PKO and WT mice (Fig. 6Ai–iii). KO mice displayed a decreased heart rate (Fig. 6B(i); WT =512 ± 24; PKO = 471 ± 25; KO = 429 ± 16 bpm; *P* < 0.05), cardiac dilatation as evidenced by an increased left ventricular (LV) end diastolic diameter (Fig. 6B(ii); WT= 2.76 ± 0.08; PKO = 2.80 ± 0.08; KO = 3.30 ± 0.18 mm; *P* < 0.05) and a proportionally increased LV systolic diameter (Fig. 6B(iii); WT = 1.30 ± 0.06; PKO = 1.28 ± 0.08; KO = 1.65 ± 0.16 mm; *P* < 0.05). LV free wall thickness (diastolic and systolic) and fractional shortening (an index of contractility), however, did not differ between genotypes. Echocardiograph pulse-wave Doppler measurements (Fig. 6C and D) revealed both a reduced E wave (early diastolic LV filling) velocity (Fig. 6D(i); WT = 78.1 ± 6.0; PKO = 79.8 ± 3.6; KO = 63.8 ± 2.2 cm.s ^−^ ^1^; *P* < 0.05;) and reduced A wave (an index of LV filling via atrial contraction) velocity (Fig. 6D(ii); WT = 53.9 ± 6.2; PKO = 59.5 ± 3.4; KO = 44.8 ± 3.1 cm.s ^−^ ^1^; *P* < 0.05) in KO mice compared to WT and PKO mice. The ratio of E:A (an index of how well the LV fills; diastolic function) was not different between groups (Fig. 6D(iii); WT = 1.51 ± 0.09; PKO = 1.36 ± 0.06; KO = 1.45 ± 0.07; *P* > 0.05). Overall, the data suggest that PKO do not display the cardiac phenotypes seen KO mice, suggesting that these defects arise in the CNS.

### Normal skeletal muscle morphology and innervation in PKO mice

**Figure 7.**
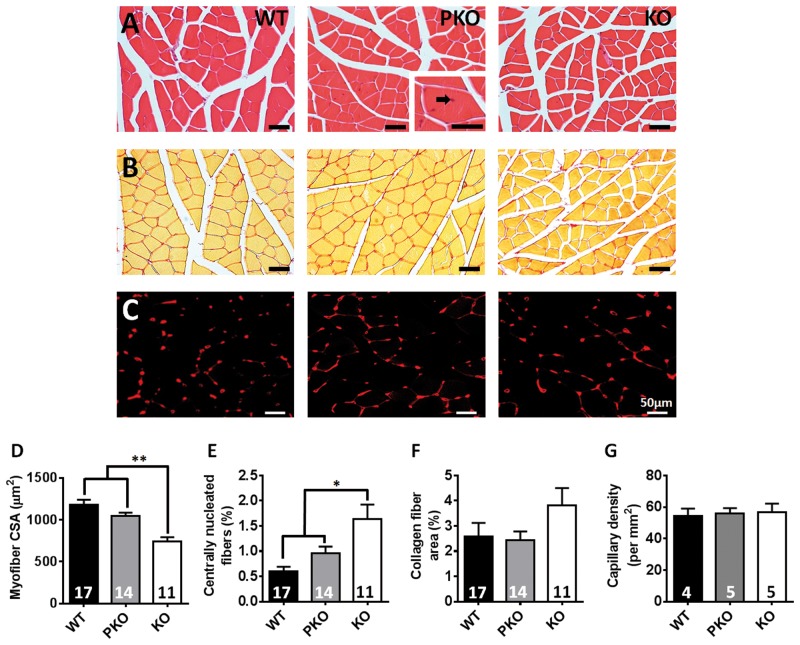
No significant muscle abnormalities in PKO mice. Representative images of gastrocnemius muscle cross section **(A)** stained with haematoxylin and eosin (H&E) for measurement of myofiber cross sectional area; **(B)** stained with picrosirius red for measurement of collagen fibers; and **(C)** immunolabelled with an antibody against the endothelial cell marker Griffonia simplicifolia lectin I (red) for measurement of capillary density. Black arrow indicates a centrally-located nucleus. Graphs show **(D)** myofiber cross sectional area in µm^2^**(E)** the proportion of fibers with a centrally-located nucleus (**F**) the proportion of the section composed of collagen fibers and (**G**) the capillary density per mm^2^. Data are plotted as mean ± SEM. Groups were compared using one-way ANOVA with Tukey’s *post hoc* comparisons. *P < 0.05, **P < 0.01.

Altered morphology of skeletal muscle including reduced fibre diameter, was recently reported in *Mecp2* KO mice (23). Histological examination of the gastrocnemius muscle in the current study showed a similar pattern of reduced fibre cross sectional area in KO mice compared to WT and PKO (Fig. 7A and C; WT = 1177 ± 64; PKO = 1045 ± 42; KO = 740 ± 53 µm^2^; *P* < 0.01) and a mildly enhanced proportion of centrally nucleated fibres (Fig. 7A and D; WT = 0.60 ± 0.09; PKO = 0.96 ± 0.13; KO = 1.64 ± 0.29%; *P* < 0.05), a putative marker of myofiber regeneration. No differences were observed between WT and PKO mice. Sections were also stained for collagen but revealed no difference between genotypes (Fig. 7B and E; WT = 2.58 ± 0.54; PKO = 2.43 ± 0.34; KO = 3.81 ± 0.69%; *P* > 0.05), consistent with previous reports (23). We also observed no difference in muscle capillary density between genotypes (Fig. 7G; WT = 54.2 ± 4.8; PKO = 55.9 ± 3.4; KO = 46.6 ± 5.4 capillaries per mm^2^; *P* > 0.05). Analysis of neuromuscular junctions revealed normal innervation of skeletal muscle fibres by innervating axons from lower motor neurons in PKO mice, with no evidence for denervation or abnormal junction morphology observed (Supplementary Material, Fig. S3).

### PKO mice show characteristic RTT-like bone phenotypes

**Figure 8.**
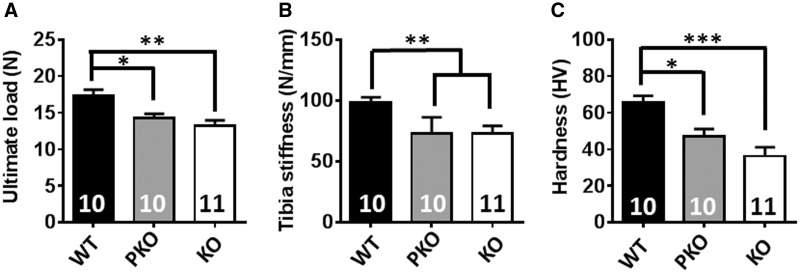
PKO mice show characteristic RTT-like bone phenotypes. Three-point bending test reveals reduced **(A)** ultimate load and **(B)** stiffness of tibia in PKO and KO mice compared to WT. **(C)** Microindentation test in polished femur reveals significantly reduced cortical bone hardness in PKO and KO mice when compared with WT controls. Plots show mean ± S.E.M. Numbers of animals per genotype are shown within each bar. Groups were compared using one-way ANOVA with Tukey’s *post hoc* comparisons. ***P < 0.001, **P < 0.01, *P < 0.05.

Spinal deformity (scoliosis and kyphosis) and other skeletal anomalies including early osteoporosis, osteopenia, bone fractures and deformities are commonly observed in females with RTT (3–5,52–54). In addition, studies in *Mecp2* KO mice have shown marked biochemical and biomechanical defects in bone tissue (21,22). To test directly whether these bone defects are central or peripheral in origin we conducted biomechanical testing of long bone samples. Functional tests carried out on tibia revealed a reduced ultimate load and stiffness in KO and PKO mice compared to WT controls (Fig. 8A and B, ultimate load: WT = 15.83 ± 0.56; PKO = 13.93 ± 0.85; KO = 12.22 ± 0.67 N; Stiffness: WT = 97.1 ± 4.0; PKO 73.31 ± 4.1; KO = 72.9 ± 6.4 N/mm; *P* < 0.05). Further biomaterial testing revealed a similar pattern in femur, with a reduced hardness of cortical bone in KO and PKO mice compared to WT littermate controls (Fig. 8C; WT = 64.7 ± 3.4; PKO = 47.0 ± 4.1; KO = 36.3 ± 4.9 HV; *P* < 0.05). Strikingly our results suggest a similarly reduced strength, hardness and lower fracture threshold in both KO and PKO mice. In the case of bone, absence of MeCP2 in peripheral tissues is likely to be a primary cause of the observed defects.

## Discussion

RTT is classically considered a neurological or neurodevelopmental disorder caused by lack of MeCP2 in the nervous system (16,33,55). Recent interest in the possibility that some phenotypes originate in the periphery is based on evidence that MeCP2 is expressed in most tissues of the body, with high levels reported in the post-mitotic cells of the heart and lungs (27–29). Here we confirm that MeCP2 expression is widespread in mouse tissues and establish that expression is much higher in the brain that in any other tested tissue. Reports of comparably high levels in lung and heart are not confirmed by our study. It is likely that normalization of MeCP2 to cytoplasmic proteins, whose abundance varies according to cell type, underlies this discrepancy. We consider that normalization to histone H3, whose abundance is constant regardless of cell origin, eliminates this confounding variable.

### The nervous system is the seat of most RTT-like phenotypes

To investigate the potential role of MeCP2 in non-neuronal organs, we developed a line of mice in which *Mecp2* is expressed from embryonic day 8.5 within the nervous system, but is largely absent from peripheral tissues. Our results reveal that most key phenotypes observed in RTT mouse models can indeed be attributed to absence of MeCP2 in the nervous system. The importance of nervous system dysfunction as the primary origin of RTT-like phenotypes is exemplified by a lack of overt RTT-like signs in PKO animals, as determined by observational scoring (33,34,56–58), and absence of reduced survival typical of KO male animals (16,17). These findings agree with previous studies adopting a reciprocal strategy whereby selective deletion of *Mecp2* in the nervous system caused reduced survival equivalent to that seen in global KO mice (16). Together, these data show clearly that the severe/lethal effects of complete MeCP2 deficiency can be attributed to lack of MeCP2 specifically within the nervous system. Whilst there were no significant differences in severity score or bodyweight between WT and PKO over the initial 30 week trial period, equivalent to the maximal survival period for the KO mice, there were detectable but modest differences at 12 months. It is not clear whether these small effects are attributable to MeCP2 deficiency in peripheral tissues or if incomplete stop cassette deletion, leading to a small minority of MeCP2-deficient cells in the brain, is the underlying cause.

Detailed phenotyping further supports the view that the key features of RTT, including breathing (49–51), balance and gait disturbances (34,45,59) are not detected in PKO mice and therefore also reflect dysfunction within the nervous system. With respect to breathing, a number of studies report disordered GABAergic and serotonergic control of brain respiratory networks as the major cause of the disrupted breathing and apnoeas in RTT in patients and mouse models (60–63). One study, however, reported that global KO of *Mecp2* caused increased apnoeas in response to hypoxia induced hyperventilation (48), whereas this effect was not seen in nervous system-specific *Mecp2*-KO mice, suggesting a non-neuronal role for MeCP2 in respiratory function. We did not specifically assess the response of mice to hypoxia, but under baseline conditions, we observed a complete rescue of apnoeas and episodic breathing in PKO mice. Our results therefore confirm that these stereotypic RTT-like features, at least under resting conditions, are due to nervous system dysfunction.

Histopathological and blood serum biochemical screens also showed few differences between genotypes confirming a lack of widespread and overt tissue pathology as a result of MeCP2 deficiency (16). The vast majority of organs showed no signs of gross structural or pathological changes with the exception of the kidney where there was some evidence of tubular vacuolation in both PKO and KO mice, a feature often associated with lipid accumulation and disordered lipid regulation (64,65). Previous observations in *Mecp2* KO mice (24) and in RTT patients (25) have suggested that the absence of MeCP2 leads to altered cholesterol biosynthesis and an increase in serum cholesterol levels. More recently, mice in which *Mecp2* was selectively deleted within the liver displayed fatty liver and an elevation in serum cholesterol (26). This is in contrast to the current study where no significant differences in serum cholesterol levels were observed across the genotypes. It is possible that this discrepancy results from background strain-specific differences, as altered cholesterol metabolism was not reported in all *Mecp2* knock-out lines (24). It is unlikely that our failure to detect differences is affected by the reported altered regulation of genes involved in lipid uptake and regulation in nestin-cre mice (37), as this transgene is present in both PKO and control mice. It is notable in this context that we did not observe the reported weight differences between WT animals with or without the nestin-cre transgene (Supplementary Material, Fig. S2), suggesting that in our lines any effects are minimal.

A recent study in mice demonstrated structural alterations in muscle fibre cross sectional area in *Mecp2* KO mice, an effect not seen in mice in which *Mecp2* is selectively deleted in skeletal muscle (23). The results of the current study support these findings and suggest that the structural abnormalities in muscle fibres seen in the KO mouse may be a consequence of disrupted nervous system function such as aberrant skeletal muscle innervation. However, we observed no difference in the structural innervation of the neuromuscular junction or any evidence of abnormal junction morphology in these mice. We also looked for evidence of disorganisation and fibrosis in muscle tissue sections from mice in the current study by staining for collagen, but found no differences between genotypes.

### Evidence for RTT-like phenotypes that arise outside the nervous system

Although the nervous system is the primary source of defects in the mouse model of RTT, our data suggest that peripheral MeCP2 deficiency leads to phenotypes, including pronounced exercise fatigue and defective bone properties. Several variables implicit in the experimental design could potentially affect interpretation of peripheral phenotypes. Firstly, it is important to ensure that *Mecp2* is activated in the vast majority of nervous system cells by deletion of the stop cassette. Our observation that levels of MeCP2 in PKO brain and spinal cord are closely similar to WT (∼90%) support this, as does immunofluorescence analysis of MeCP2 in specific CNS regions including the spinal cord. Nevertheless, it could be argued that some moderate phenotypes are due to the deleterious effect of the small residual population of MeCP2-negative nervous system cells. We note, however, that phenotypes that require sophisticated brain function, including balance and innate nest building behaviour, were indistinguishable between PKO and WT mice. Furthermore, previous studies using a less efficient cre-based strategy that resulted in 60–80% recombination in the nervous system resulted in functional reversal of neuronal plasticity and a range of motor phenotypes (33,34). This supports the interpretation that defects seen in the PKO mice most likely originate in MeCP2-deficient peripheral tissues. The finding that bone defects are equally severe in the PKO mice, which have almost WT levels in the brain, and in KO mice lacking almost all brain MeCP2 strongly argues that this phenotype originates outside the nervous system.

A second potential source of confounding effects would arise if the absence of MeCP2 in nervous system prior to nestin-cre mediated activation of the gene has long-term phenotypic consequences. This seems unlikely as nestin expression commences early in development (day 8 of embryogenesis) and is pervasive in neuronal progenitors, whereas MeCP2 expression is low at this time, only increasing dramatically in the nervous system after birth when most neurogenesis is complete (29). Accordingly, the phenotypic effects of MeCP2 absence do not become apparent until several weeks after birth. These observations suggest that MeCP2 deficiency in early embryogenesis is probably phenotypically neutral. A third consideration affecting interpretation of the results is the possibility that a small proportion of MeCP2-positive cells in non-CNS tissues masks phenotypes that would have been apparent if these tissues were truly null. The data suggest less than 10% of normal levels of MeCP2 remains peripherally. This level of MeCP2 in the brain would cause a severe Rett-like phenotype, as even a 50% reduction gives detectable Rett-like features (66,67). We found that recombination efficiency in the kidney was higher and therefore more cells probably retain MeCP2 expression. In addition, previous studies have shown nestin expression in the vascular wall (68). Our ability to detect kidney phenotypes or those impacted by vasculature function may therefore be somewhat compromised.

A particularly robust finding in this study is the marked reduction in exercise capacity and vulnerability to fatigue displayed by PKO animals. Whilst levels of spontaneous activity in the open field test were only moderately reduced in comparison to WT, when animals were challenged with more intensive tasks (such as the elevated treadmill) the deficit was more pronounced, with an almost 50% decrease in performance compared to WT. Although less severe than the deficit in KO mice, the finding nevertheless suggests an exercise fatigue phenotype that may be a consequence of peripheral MeCP2 deficiency. Importantly, inertia and reluctance to move are widely reported in *Mecp2* KO mice (33,69). So far we have been unable to attribute fatigue to a specific organ system. We consider cardiorespiratory dysfunction to be an unlikely source as PKO mice did not differ from WT when assessed for a range of cardiac (heart rate, diastolic filling parameters and left ventricular systolic and diastolic dilation) and respiratory measures. Muscle morphology and neuromuscular innervation were also normal in PKO mice. It is possible that future studies assessing cardiac and respiratory responses under exercise conditions, or assessing more subtle aspects of muscle physiology and metabolism, may identify peripheral MeCP2-mediated contributions to the observed fatigue phenotype.

An important unanticipated finding of our study is that functional bone phenotypes seen in RTT-mice are due to a loss of MeCP2 in peripheral tissue. A number of recent reports have shown abnormalities in the biomechanical and structural properties of bone as well as biochemical differences and altered osteoblast activity in *Mecp2* KO mice (21,22,70). It was unclear from these studies, however, whether the primary cause was a local deficiency of MeCP2 or a secondary consequence of MeCP2 deficiency within the nervous system. Results from PKO animals in the current study suggest that biomechanical and biomaterial defects are in fact primarily peripheral in origin as KO and PKO animals both show similar levels of dysfunction when compared to WT animals. This is an important finding as skeletal anomalies, such as early osteoporosis and low energy fractures, are well documented in RTT patients (4,5,53,54,71). Treatments targeted solely at the nervous system are unlikely to ameliorate these effects. It is not clear from the current study whether the bone phenotypes result from MeCP2 deficiency in bone cells *per se*, or whether they are secondary to MeCP2-related defects in other peripheral tissues. Recent evidence, however, has shown dysfunction in MeCP2-deficient osteoblasts compared to wild-type cells suggesting the phenotype may be primary in origin (70). The PKO mouse model described here is likely to be an important tool in further dissecting the physiology of these clinically relevant effects.

## Materials and Methods

### Animals

Cohorts of male PKO, WT, WT-Cre and KO mice were taken from litters produced by mating hemizygous *Nestin-Cre* males (35) (B6.Cg-Tg(Nes-cre)1Kln/J, Jackson Laboratories stock no. 003771) with heterozygous *Mecp2^+/Stop^* females (33) (B6.129P2-*Mecp2tm2Bird*/J, Jackson Laboratories stock no. 006849). *Nestin-Cre* males were on an inbred C57BL6/J (C57) genetic background and *Mecp2^+/Stop^* females came from a breeding colony on a mixed C57BL6/J, CBA/CaOlaHsd (C57/CBA) genetic background. Initiated by crossing C57/CBA F1 WT males and *Mecp2^+/Stop^* females, the colony had been maintained over many generations by crossing *Mecp2^+/Stop^* females from the colony with WT C57/CBA F1 males, thus maintaining an approximately equal contribution from each genetic background strain.

Experimental cohorts of *Mecp2^Stop/y^* (KO); *Mecp2^Stop/y^, Nestin-Cre* (PKO); *Mecp2^+/y^* (WT) and *Mecp2^+/y^, Nestin-Cre* (WT-Cre) littermates were genotyped by PCR as previously described (33).

All animals were housed with littermates and maintained on a 12 h light/dark cycle and given access to food and water ad libitum. Experiments were carried out in accordance with the European Community Council Directive (86/609/EEC) and project licences with local ethical approval under the UK Animals (Scientific Procedures) Act (1986).

### Southern blot

Genomic DNA was prepared from tissues and Southern blotted as previously described (33). Briefly, Southern blots of *Eco*RI/*Nco*I double-digested genomic DNA were probed with a 1.1 kb *Hin*dIII fragment carrying the part of the mouse MeCP2 ORF contained in exon 4. The percentage recombination of the *Mecp2^Stop^* allele was quantified by scanning radiolabelled blots using a Typhoon PhosphorImager (GE Healthcare) and determining the percentage of the total signal (Stop + Deleted bands) contained in the Stop band using ImageQuant software (GE Healthcare).

### Western blot

Whole-cell homogenates of various tissues were prepared for western blotting by homogenizing in NE1 buffer (20 mM HEPES pH 7.9, 10 mM KCl, 1 mM MgCl_2_, 0.1% Triton X-100, 20% glycerol, 0.5 mM DTT, protease inhibitors cocktail (Roche)) using an Ultra-Turrax T25 homogeniser, followed by incubation with 1000U/ml Benzonase (Sigma) for 15 min at room temperature. An equal volume of 2 x SDS-PAGE sample buffer was added and the samples were boiled, snap frozen and boiled again before centrifuging for 5 min and taking the supernatant. Samples were run on Bio-Rad TGX gradient gels (4–20%) and blotted onto 0.2μm nitrocellulose membrane by an overnight wet transfer in 25 mM Tris, 192 mM glycine at 25 V and 4 °C. Membranes were blocked in 5% skimmed milk powder in PBS before incubating at 4 °C overnight with primary antibodies (anti-MeCP2 mouse monoclonal, Sigma M7443, 1:1,000 and anti-histone H3 rabbit polyclonal, Abcam ab1791, 1:10,000). Antibodies were diluted in 5% skimmed milk powder in PBS + 0.1% TWEEN-20. After washing in PBS, membranes were incubated at RT for 2 h with IR-dye secondary antibodies (IRDye 800CW donkey anti-mouse, IRDye 680LT donkey anti-rabbit, LI-COR Biosciences) diluted at 1:10,000 and then scanned using a LI-COR Odyssey machine. Images were quantified using Image Studio Lite software (LI-COR Biosciences). MeCP2 levels were normalised to histone H3 for each sample and values from each sample were expressed as a percentage of the whole brain reference sample from the same gel to enable comparison of MeCP2 levels between different gels and different tissues.

### Immunohistochemistry

Mice were humanely euthanized by intraperitoneal injection of a lethal dose of Euthatal and transcardially perfused with 4% paraformaldehyde in 0.1 M phosphate buffer solution. Brain, lung, heart and gastrocnemius muscles were dissected, post-fixed, and dehydrated through increasing concentrations of ethanol and amyl acetate, before being embedded in paraplast. Sections (5 µm) were collected on APES coated slides and dried overnight at 37 °C. Sections were then deparaffinised and rehydrated by Histo-Clear and decreasing concentrations of alcohol, before being incubated in 0.1 M sodium citrate buffer (pH 6.0) for 2 × 5 min in a 950 watt microwave oven at full power for antigen retrieval.

#### Anti-MeCP2

In order to block the endogenous peroxidase and prevent non-specific binding, sections were first incubated in 3% hydrogen peroxide (VWR) in methanol for 30 min, followed by a 1 h incubation with 5% bovine serum albumin (Sigma) and 20% normal goat serum (Sigma) in 0.05 M TBS at room temperature. The sections were then incubated with the primary antibody (mouse anti-MeCP2, Sigma, 1: 250) overnight at 4 °C, rinsed with 1% TBS/Tween-20 and incubated with the secondary antibody (biotinylated goat anti-mouse, Jackson, 1:200) for 1 h at room temperature. Sections were then treated with Avidin-Biotin-Complex (Vectastain ABC kit, Elite-PK-6100 Vector Labs) for 30 min followed by incubation with 3, 3-diaminobenzidine (DAB) (Vector Laboratories). Finally, sections were stained with Mayer’s hematoxylin before being dehydrated and mounted with DPX. Images were captured using a CCD camera (Axiocam HRc, Zeiss, Germany) mounted on the light microscope (Eclipse 800, Nikon, Japan).

#### Capillary density measurements

Sections were washed in 0.3 M PBST (3 × 10 min) and incubated with 5% NGS in 0.3 M PBST for 1 h at room temperature. Sections were then immunolabelled with rhodamine-labelled Griffonia simplicifolia lectin-1(GSL-1; Vector Laboratories RL-1102; 1:100) and incubated overnight at 4 °C. Sections were rinsed with 0.3 M PBST (3 × 10  min) and mounted with vectashield. Images were collected using a BioRad MRC 1000 laser scanning confocal microscope, 40X oil. The capillary density (number of capillaries per mm2) was determined using ImageJ, with a minimum of five non overlapping areas used for each sample.

### Weight measurements and severity scoring

Each week all animals were weighed and scored using an observational severity scoring system, as previously described (33). Briefly, animals were observed for each of six signs (tremor, breathing, hind-limb clasping, gait, mobility, and overall general condition) related to the Rett-like phenotype and given a score of either 0 (absent i.e. as wild-type), 1 (present), or 2 (severe); these scores are then added to give an overall aggregate severity score out of 12 for each mouse. Scoring was carried out blind to genotype. Animals were culled according to previously described criteria (33) - scored 2 for tremor, breathing, or general condition, or losing >20% of their bodyweight over a period of 1 week. Animals culled in this way were treated as having died in the survival analysis.

### Blood biochemistry

Mice were euthanized by CO_2_ inhalation and blood samples acquired via terminal cardiac puncture. Samples were quickly transferred to lithium heparin coated polypropylene tubes to prevent clotting and transported within the hour to a specialised small animal clinical pathology lab for biochemical analysis.

### Histopathological analysis

An array of tissue samples were collected and embedded in paraffin for sectioning and haematoxylin and eosin (H&E) staining. Tissue sections were then assessed by a qualified veterinary pathologist, blind to genotype.

### Open field

Locomotor function and exploratory behaviours were investigated using an open-field test. Mice were placed in the centre of a 60 cm diameter circular arena and allowed to explore freely for 10 min, during which time the arena was imaged using an overhead digital camera and the animal was detected and tracked using Ethovision 3.1 tracking software (Noldus Inc, Leesburg, VA). A number of movement related parameters were calculated. The test was carried out twice for each mouse on consecutive days and the mean of these replicates was used as the data point for each mouse for each parameter.

### Nest building

Home cage nest quality was assessed using a previously described scoring system (40). Briefly, mice were individually caged overnight and supplied with 8 g of shredded biodegradable paper strips as a nesting material, distributed evenly over cage floor. Next morning nest quality was assessed using a five point scoring system, which rated nests based on the formation of a central nest hollow with surrounding walls (Fig. 2.1). The lowest score of zero was given if the nesting material was undisturbed, and no signs of interaction or manipulation were seen. The maximum score of five was given when the mouse had constructed a fully formed nest, with walls completely enclosing a central hollow In order to assign a score, the lowest point of the nest was identified and scored with an additional 0.25 being added to the score for each quarter of the nest that had a higher wall. Scoring was carried out by two independent scorers blind to genotype.

### Gait analysis

Gait analysis was carried out using the DigiGait imaging system (Mouse Specifics, Boston, MA) as previously described (26). Data were collected at a running speed of 10 cm/s.

### Balance beam

Mice were tested for a combination of balance and coordination using an inclined balance beam. Beams were either 5 mm or 11 mm wide and the time taken to traverse a 50 cm span was recorded. Six trials on each beam were carried out, three trials per day on two consecutive days, and the mean of all six trials was used as the data point for each animal. Animals that had not crossed the beam within 1 min were given the maximum score of 60  s.

### RotaRod

Motor learning and coordination were investigated using a 5-lane accelerating rotarod (UGO Basile, Italy). The rotation speed was set to provide a gradual acceleration from 1rpm to 45 rpm over a period of 5 min. Mice were scored for how long they remained on the rod without falling or passively rotating. Three trials each day, on two consecutive days, were carried out and the mean of all 6 trials was used as the data point for each animal.

### Exercise tolerance

Exercise capacity of the mice was investigated using an accelerating elevated treadmill. A mild aversive stimulus (electric shock) was applied at the base of the treadmill in order to ensure the mice performed to maximal physiological capacity. Mice were placed on the treadmill at an initial speed of 10 cm/s and the speed was increased by 2 cm/s every 2 min until maximum endurance was reached and the mice stopped running. At this point the trial was terminated and the time was recorded (72).

### Whole-body plethysmography

Respiratory phenotype was determined in conscious and unrestrained mice using a whole body plethysmography apparatus (EMMS, Bordon, U.K.). Mice were placed inside a Plexiglas chamber and left for 20 min to become used to the environment after which their breathing was monitored for 30 min. To account for differing levels of movement and grooming between groups, only data from periods in which the mice remained at quiet rest was analysed. A continuous bias airflow supply allowed the animal to be kept in the chamber for extended periods of time. Pressure changes caused by alterations in the temperature and humidity of the air as it enters and leaves the subjects’ lungs were detected by a pressure transducer, and a respiratory waveform representing the breathing pattern of the animal was produced. The waveform was then exported and analysed using pClamp 10.2 (Molecular Devices Inc., California, USA). Respiratory waveforms were analysed for breathing frequency, frequency variability (using the coefficient of variability of the waveforms) and the frequency of apnoeas (expiratory pauses more than three respiratory cycles in length).

### Echocardiography

Animals were anaesthetised by inhalation of 4% isofluorane gas (Isoflo, Abbott Laboratories, USA) delivered in O_2_ at 1.5 l.min ^−^ ^1^ in an induction chamber until loss of righting reflex. The animals were then maintained on 1.5–2.0% isofluorane gas delivered in 1.0 l.min ^−^ ^1^ O_2_ delivered by face mask. Fur was clipped from the ventral thorax, cleaned with chlorhexidine spray and warmed ultrasound gel applied to enhance acoustic transmission. B-mode and M-mode echocardiographic measurements of the left ventricular chamber were taken with a 15 MHz neonatal cardiac probe (Acuson Sequoia 512, Siemens U.K.) in the parasternal short-axis (transverse) view at the level of the papillary muscles. Dimensions recorded were: left ventricular end diastolic diameter (LVEDD), left ventricular end systolic diameter (LVESD), end diastolic posterior wall thickness (PWD), end systolic posterior wall thickness (PWS) and the change in LV diameter from diastole to systole (fractional shortening; FS). Blood velocity through the mitral valve was measured in the apical four-chamber view using colour Doppler to identify the atrio-ventricular blood flow and using pulse-wave Doppler to measure velocity. Parameters recorded were; E wave (first component of LV filling due to relaxation of the LV chamber), A wave (second component of LV filling due to atrial contraction) and E:A ratio (an indicator of LV diastolic function).

### Structural analysis of muscle

Gastrocnemius muscles were dissected and immediately fixed in 4% paraformaldehyde in 0.1 M phosphate buffer solution for 24 h, before being processed and paraffin embedded. Cross-sections (7µm) were then stained, either with Haematoxylin and Eosin to measure myofiber cross-sectional area, or with Picrosirius red to measure intramuscular connective tissue. Bright field images were then captured from non-overlapping fields of the entire section and analysed using ImageJ (NIH, USA). The myofiber cross-sectional area was measured by manually tracing the circumference of each fibre following a previously described protocol (73). To quantify connective tissue levels, images were converted to an RGB stack and the green channel was selected as it produces higher contrast. Red-stained collagen was identified by applying thresholding of grayscale to measure the percentage of collagen in relation to the total field area.

### Neuromuscular junction analysis

Mice were culled by inhalation of isoflurane. The lower limb lumbrical and interscutularis muscles were dissected out and fixed in 4% paraformaldehyde for 30 min. Neuromuscular junctions were then immunolabelled for the pre-synaptic proteins SV2 (synaptic vesicle) and 2H3 (neurofilament), and post-synaptic AChRs as previously described (74,75). For each individual muscle (lower limb lumbrical and interscutularis), counts were performed on 40 neuromuscular junctions. Representative images were acquired on a Zeiss LSM 710 confocal microscope.

### Bone biomechanical tests

Biomechanical testing of tibia and micro-hardness testing of polished femur section was conducted as described previously (21). Briefly, mouse tibial shafts were scanned by micro-computed tomography (SKYSCAN^®^1172/A μCT Scanner, Bruker, Belgium) before being subjected to a three-point bending test for stiffness and strength of cortical bone using a Zwick/Roell z2.0 testing machine (Leominster, UK) with a 100 N load cell. Tibias were placed between supports with 8 mm separation and load applied at the mid-point between the supports at a rate of 0.1 mm.s ^−^ ^1^ until fracture occurred. Data were analysed to determine values of stiffness, ultimate load and Young's modulus (21). The material properties of the bone were assessed using a micro-indentation hardness test performed on segments of polished femur located in the distal mid-shaft region. Micro-hardness testing was performed using a Wolpert Wilson Micro-Vickers 401MVA machine (UK), with an applied load of 25 g for 100 s. Each sample was tested at seven points and the Vickers hardness number (HV) calculated (21).

## Statistical Analysis

Group data are expressed as mean ± SEM throughout the text. Groups were compared using Kruskal-Wallis tests with Dunn’s *post hoc* analysis (for survival and composite severity score), unpaired t-test (bodyweight) and one-way ANOVA with Tukey’s *post hoc* analysis (all other tests). Analysis was carried out using Minitab 17.0 (Minitab, USA). Significance was accepted at *P* < 0.05.

## Supplementary Material

Supplementary Material is available at *HMG* online.

## Supplementary Material

Supplementary Data
