# Supplementary material for: Exclusive expression of MeCP2 in the nervous system distinguishes between brain and peripheral Rett syndrome-like phenotypes
Source: Hum Mol Genet. 2016 Aug 9;25(20):4389–404. doi: 10.1093/hmg/ddw269 (PMC5886038; doi:10.1093/hmg/ddw269)
Supplement: Supplementary Data [file ddw269_supp.docx]

**
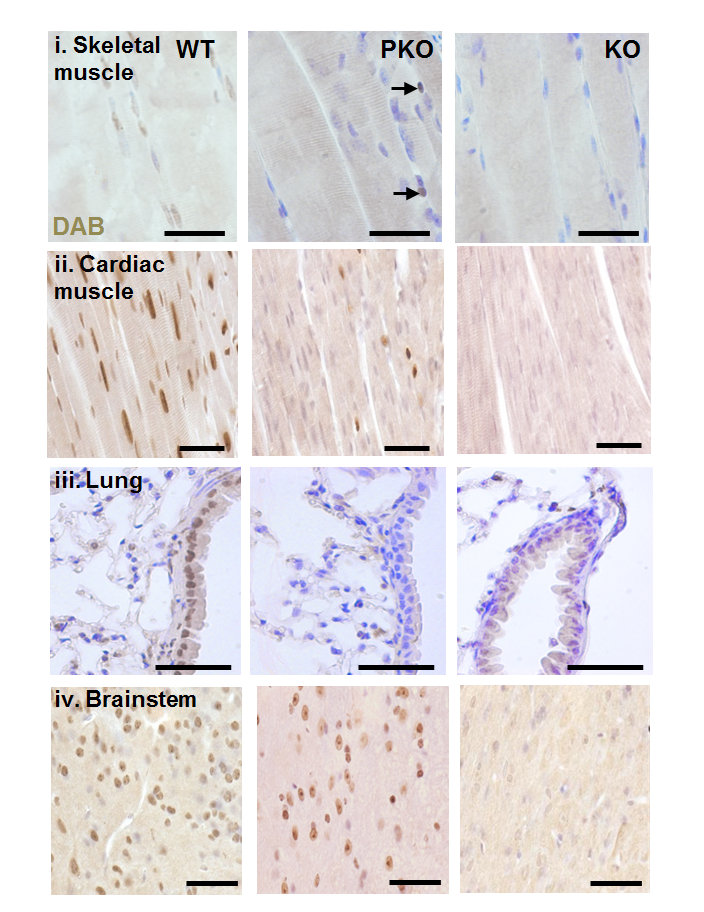
**

**Supplementary Figure 1. Distribution of MeCP2 in brain and peripheral tissues**

Representative bright field images showing anti-MeCP2 peroxidase-labelled nuclei (brown) in (i) skeletal muscle, (ii) cardiac muscle, (iii) lung and (iv) brainstem. Arrows in (i) indicate very sparse peroxidase product labelling of nuclei. Scale bars: 40µm.

**
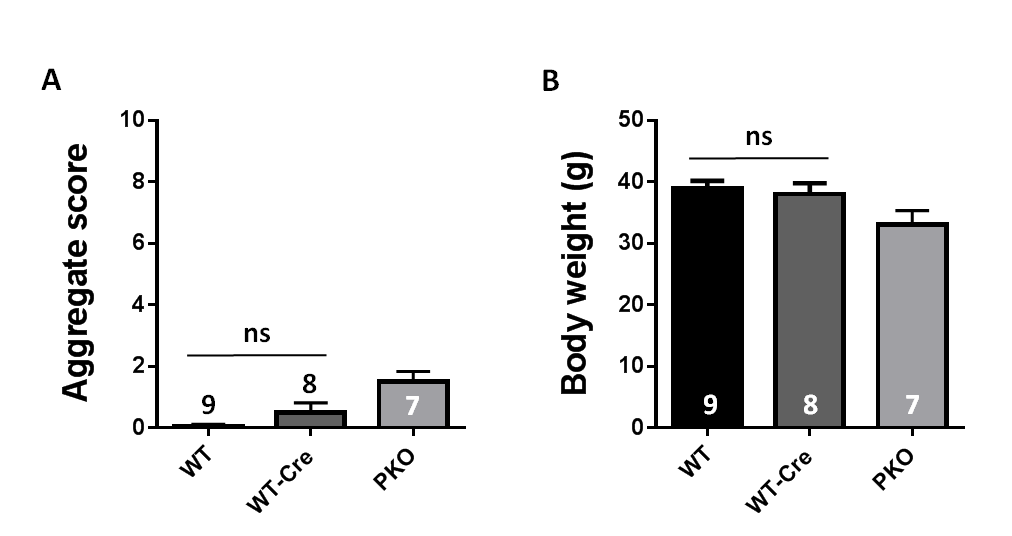
**

**Supplementary Figure 2.** No significant difference in aggregate score and body weight between WT and WT-cre mice at 52 weeks. Plots showing (A) aggregate phenotype severity (B) body weight at 52 weeks of age. Results show mean ± SEM. No significant difference was seen between WT and WT-cre mice (one-way ANOVA with Tukey’s post hoc analysis). Number of animals per genotype are shown within each bar.

**
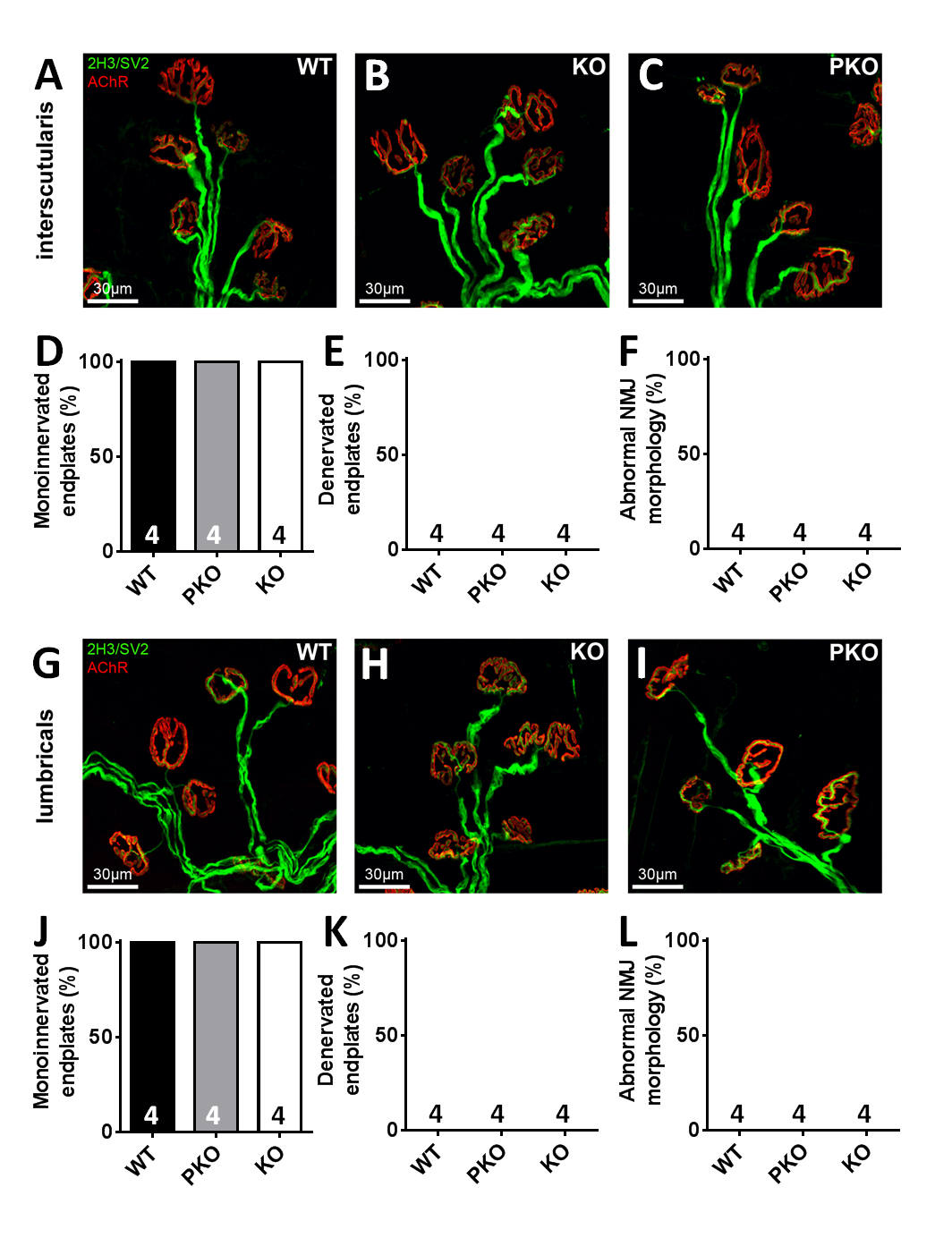
**

**Supplementary Figure 3. Neuromuscular junction assessment in WT, KP and PKO mice.** (A-C) Representative immunofluorescent images showing morphology of NMJ from interscutularis muscle from WT, KO and PKO mice respectively. Bar charts illustrating percentage of (D) monoinnervated NMJs , (E) denervated NMJs and (F) NMJs with abnormal morphology (neurofilament accumulation, changes in shape or size). G-L Equivalent data from interscutularis muscle. Number of animal per genotype is shown. Red shows α-bungarotoxin labelling of postsynaptic nicotinic acetyclcholine receptors (AChRs) and green shows presynaptic anti-SV2 (synaptic vesicle) and 2H3 (neurofilament) labelling. Scale bars: 30 µm.

**Supplementary Table 1. Blood biochemistry results**

|  | **WT PKO KO** | | |
| --- | --- | --- | --- |
| **Sodium (mmol/l)** | 151.7 ± 0.6 | 153.4 ± 0.8 | 153.1 ± 1.8 |
| **Potassium (mmol/l)** | 7.22 ± 0.22 | 7.83 ± 0.33 | 8.43 ± 1.01 |
| **Sodium:Potassium ratio** | 21.29 ± 0.70 | 20.86 ± 0.40 | 18.78 ± 2.04 |
| **Chloride (mmol/l)** | 110.9 ± 0.5 | 109.4 ± 0.5 | 108.1 ± 1.3 * |
| **Calcium (mmol/l)** | 2.48 ± 0.02 | 2.49 ± 0.04 | 2.48 ± 0.07 |
| **Phosphate (mmol/l)** | 2.64 ± 0.16 | 2.95 ± 0.10 | 3.81 ± 0.68 * |
| **Urea (mmol/l)** | 10.72 ± 0.58 | 10.80 ± 0.52 | 20.60 ± 9.40 |
| **Creatinine (µmol/l)** | 30.50 ± 0.90 | 31.55 ± 0.86 | 41.22 ± 13.11 |
| **Cholesterol (mmol/l)** | 3.26 ± 0.09 | 3.65 ± 0.15 | 3.01 ± 0.23 |
| **Triglyceride (mmol/l)** | 2.53 ± 0.27 | 2.54 ± 0.33 | 2.20 ± 0.25 |
| **Total bilirubin (µmol/l)** | 2.57 ± 0.31 | 4.55 ± 1.12 | 3.14 ± 0.67 |
| **ALK phos (U/l)** | 245.9 ± 12.3 | 247.0 ± 28.9 | 297.4 ± 30.6 |
| **AST (U/l)** | 127.3 ± 15.5 | 164.0 ± 55.85 | 201.7 ± 43.3 |
| **ALT (U/l)** | 44.79 ± 7.27 | 44.67 ± 4.47 | 153.0 ± 73.11 |
| **GGT (U/l)** | Not detectable | Not detectable | Not detectable |
| **Total protein (g/l)** | 53.64 ± 0.78 | 56.09 ± 1.16 | 58.11 ± 1.33 * |
| **Albumin (g/l)** | 29.79 ± 0.54 | 31.27 ± 0.45 | 33.00 ± 1.08 * |
| **Globulin (g/l)** | 23.86 ± 0.43 | 24.82 ± 0.80 | 25.11 ± 0.59 |
| **Albumin: Globulin ratio** | 1.25 ± 0.03 | 1.27 ± 0.03 | 1.32 ± 0.04 |

Arterial blood was sampled at 15 weeks for biochemical analysis. Data show mean values ± S.E.M. Groups were compared using one-way ANOVA with Tukey’s post hoc analysis. Group sizes were WT = 14, PKO = 11, KO = 9. * *p* < 0.05 compared to WT.

**Supplementary Table 2. Histopathological screening results**

|  | **WT 1** | **WT 2** | **WT 3** | **PKO 1** | **PKO 2** | **PKO 3** | **KO 1** | **KO 2** | **KO 3** |
| --- | --- | --- | --- | --- | --- | --- | --- | --- | --- |
| **Heart** | - | - | - | - | - | - | - | - | - |
| **Lungs** | - | - | - | - | - | - | - | - | - |
| **Liver** | - | - | ­🟋 | ­🟋 | - | - | ­🟋 | ­🟋 | - |
| **Salivary glands** | - | - | - | - | - | - | - | - | - |
| **Mandibular lymph nodes** | - | - | - | - | - | - | - | - | - |
| **GI system** | - | - | - | - | - | - | - | - | - |
| **Pancreas** | - | - | - | - | - | - | - | - | - |
| **Kidneys** | - | - | - | + | + | + | + | - | + + |
| **Adrenal gland** | - | - | - | - | - | - | - | - | - |
| **Spleen** | - | - | - | - | - | - | - | - | - |
| **Skin dorsal** | - | - | - | - | - | - | - | - | - |
| **Skin inguinal** | - | - | - | - | - | - | - | - | - |
| **Thymus** | - | - | - | - | - | - | - | - | - |
| **Mesenteric lymph nodes** | - | - | - | - | - | - | - | - | - |
| **Femur & knee joint** | - | - | - | - | - | - | - | - | - |
| **Sternum** | - | - | - | - | - | - | - | - | - |
| **Cranium** | - | - | - | - | - | - | - | - | - |
| **Brain** | - | - | - | - | - | - | - | - | - |
| **Tail** | - | - | - | - | - | - | - | - | - |

H&E stained tissue sections for organs listed above were assessed for histopathological changes by a veterinary pathologist. 3 animals were examined per genotype. Results of analysis are indicated using key below.

- No gross or histopathological changes observed

🟋 Moderate to diffuse coarse cytoplasmic clearing/vacuolation

+ Very mild vacuolation in the renal tubule epithelium

+ + Moderate vacuolation in the renal tubule epithelium

**Supplementary Table 3. Treadmill-based gait analysis results**

|  | **Mean Value ± S.E.M**  **WT PKO** | |
| --- | --- | --- |
| **Stride Length (cm)** | 4.76 ± 0.09 | 4.83 ± 0.10 |
| **Stance Width (cm)** | 2.23 ± 0.09 | 2.33 ± 0.12 |
| **Step Angle (deg)** | 55.59 ± 2.11 | 52.54 ± 1.69 |
| **Hind Limb Shared Stance Time (s)** | 0.08 ± 0.01 | 0.07 ± 0.01 |
| **Gait Symmetry** | 0.99 ± 0.01 | 0.99 ± 0.01 |
| **Overlap Distance (cm)** | 1.25 ± 0.08 | 1.45 ± 0.08 |

The table shows mean values ± S.E.M for gait parameters assessed during treadmill-based gait analysis (see reference ([1](#_ENREF_1)) for parameter definitions). Groups were compared using student’s unpaired t-test. Group sizes were WT = 10, PKO = 11.

1 Gadalla, K.K., Ross, P.D., Riddell, J.S., Bailey, M.E. and Cobb, S.R. (2014) Gait analysis in a Mecp2 knockout mouse model of Rett syndrome reveals early-onset and progressive motor deficits. *PLoS One*, **9**, e112889.
